# The Relationship of Dyadic Coping With Emotional Functioning and Quality of the Relationship in Couples Facing Cancer—A Meta-Analysis

**DOI:** 10.3389/fpsyg.2020.594015

**Published:** 2021-01-08

**Authors:** Adelina Mihaela Ştefǎnuţ, Mona Vintilǎ, Otilia Ioana Tudorel

**Affiliations:** Department of Psychology, West University of Timişoara, Timişoara, Romania

**Keywords:** cancer, dyads, quality of relationship, meta-analysis, emotional functioning

## Abstract

**Objective:** This study is a meta-analysis that considers the association between dyadic coping and emotional functioning, and between dyadic coping and the quality of the relationship as perceived by cancer patients and their life partners.

**Methods:** A systematic search was conducted in the electronic databases PsycINFO, PubMed, ScienceDirect and those peer-reviewed cross-sectional and longitudinal studies published up until April 2020 that investigated these relationships were selected.

**Results:** A total of 1,168 studies were identified, of which 10 met the inclusion criteria (*N* = 1,727 couples). These evidenced statistically significant positive relationships between common dyadic coping and emotional functioning and between common dyadic coping and the quality of the relationship as perceived by patients and their partners. There was also a statistically significant positive association between stress communication (by oneself), supportive dyadic coping (by oneself and by partner), and the quality of the relationship. In addition, a statistically significant negative association was found between negative dyadic coping (by oneself and by partner) and the quality of the relationship as perceived by patients' partners and also between negative dyadic coping (by oneself) and the quality of the relationship as perceived by patients.

**Conclusions:** The results suggest the existence of a significant association between dyadic coping and emotional functioning and between dyadic coping and the quality of the relationship as perceived by members of couples facing cancer. However, these results must be interpreted with caution due to the small number of studies included in the analysis. Clinically, an understanding of the existence of such relationships is helpful for the implementation, and study of the effectiveness of, interventions aimed at improving dyadic coping in order to improve both quality of life and quality of relationship in couples where there is an oncological diagnosis.

## Introduction

Cancer is a life-threatening disease that represents one of the most difficult experiences that a person can be faced with during their life. The development of effective treatments has contributed to a gradual reduction of the taboo which previously accompanied open discussion of a diagnosis, to the investigation of the psychological and social aspects associated with the disease, and to a search for the most appropriate methods of offering support. Over time psycho-oncological research has employed a range of theoretical principles that have led to a corresponding variety of approaches.

One of the most used theoretical frameworks in psychological research in the context of cancer has been the Transactional Model of Stress and Coping proposed by Lazarus and Folkman (1984), in which social support is seen as a way of helping people cope with stress. Following this, research frequently centered on patients (Stark and House, 2000; Massie, 2004; Drageset and Lindstrøm, 2005; Thomsen et al., 2010), with their relatives being seen mainly as sources of support. However, it became clear that the chief source of support for cancer sufferers was frequently their life partners (Kim and Spillers, 2010) and that these people in their turn faced high levels of distress (Jaafar et al., 2014; Heckel et al., 2015), sometimes higher than those experienced by patients (Couper et al., 2006). This led to a recognition that cancer patients' partners too were in need of support (Northhouse and Muhammad, 2000; Jaafar et al., 2014; Heckel et al., 2015). It also became evident both that the process of coping with cancer affects both members of a partnership and that they influence one another (Li and Loke, 2014), and thus cancer began to be regarded as a “we-disease” (Kayser et al., 2007). In recent years psycho-oncological research has widened its focus from a concentration on the individual (patient or partner) toward a dyadic perspective, from individual coping to dyadic coping.

This modification means that stress and coping with stress are no longer seen as intrapsychic phenomena, but as interdependent processes experienced by the couple, ones in which cognitive evaluation, feelings and coping behaviors are shared by the two of them (Revenson et al., 2005). Professionals agreed to use the following terms in their formal language, as common ground was needed (Goian, 2004, 2010). Dyadic stress is the term used for situations, such as a cancer diagnosis, which affect both partners directly or indirectly and trigger a shared coping endeavor. Dyadic coping involves the interdependence of the partners, shared concerns, and shared purposes which stimulate a resolving of the problems together and shared activities aimed at emotional balance. Dyadic coping supplements individual coping strategies and its purpose is to restore homeostatic balance both for each individual and for the couple as such (Bodenmann, 1997, 2005).

Several models of dyadic coping have been defined, but according to Falconier et al. (2015) the only ones that do not also include individual coping strategies but only take into consideration the way the two partners show mutual support in facing stress are The Relationship-Focused Coping Model (DeLongis and O'Brien, 1990; Coyne and Smith, 1991), The Systemic-Transactional Model (Bodenmann, 1997) and The Developmental-Contextual Coping Model (Berg and Upchurch, 2007). Since the review carried out by Regan et al. (2015) demonstrates that the Systemic-Transactional Model (STM) provides the most comprehensive model for elucidating the behaviors exhibited by couples confronting cancer, it is this way of conceptualizing dyadic coping that we will be focusing on in this paper.

STM is based on the Transactional Model of Stress and Coping developed by Lazarus and Folkman, which comprehends concepts such as the perception of stress, evaluation of stress and the coping response but extends this model to the systemic dimension. Thus, following Bodenmann (2005), after one of the partners has perceived and evaluated stress, they engage in a process of verbal or non-verbal communication with the other partner. The receiving partner perceives, interprets and decodes these signals and engages in a kind of dyadic coping. Dyadic coping can take both positive and negative forms. *Positive dyadic coping* includes *supportive dyadic coping* (by oneself or by partner – by which help is given to the partner in their coping efforts in a variety of ways, such as empathetic understanding and the expression of solidarity), *delegated dyadic coping* (by oneself or by partner – by which one of the partners takes over some of the responsibilities of the other with the aim of helping them), and *common dyadic coping* (by which the two partners take action together in order to address the situation). *Negative dyadic coping* can take the form of ambivalent, hostile or superficial behaviors. Ambivalent behaviors occur when the partner offers support unwillingly, accompanying this help with an attitude that suggests that his or her contribution is not necessary. Hostile dyadic strategies consist of the fact that the partner offers support in a negative way, accompanied by distance, disinterest, sarcasm, or minimizing the seriousness of the other's stress. Superficial dyadic coping refers to the fact that the support offered is insincere, devoid of empathy.

The importance of dyadic coping for mental and physical functioning and for the functioning of the relationship has been established for a number of types of stressors (Vilchinsky et al., 2010; Duca and Turliuc, 2014; Turliuc and Rusu, 2014; Bertoni et al., 2015). As well as the present paper, the mentioned researches were interested in the relationship between dyadic coping and other psychological variables. Although there are experimental studies that considered causal relationships in which dyadic coping was involved, they were not mentioned in order not to create ambiguity. Studies have been devised to investigate dyadic coping in the context of different chronic conditions: diabetes (Johnson et al., 2013), chronic obstructive pulmonary disease (Meier et al., 2011; Vaske et al., 2015), kidney transplant (Tkachenko et al., 2019), chronic pain (Burri et al., 2017) and cancer. It has been shown that in the case of couples facing a diagnosis of breast cancer there is a positive relationship between relational mutuality and common dyadic coping and positive dyadic coping, both for patients and their partners, and also a negative relationship between relational mutuality and the avoidance of dyadic coping, a negative dyadic coping style (Kayser and Acquati, 2019). Additionally, for couples facing breast cancer, it has been shown that levels of depression experienced by both partners reduce in direct proportion to the extent to which they engage in common dyadic coping (Rottmann et al., 2015). Both for patients with metastatic breast cancer and their partners the exercise of negative dyadic coping was associated with higher levels of distress (Badr et al., 2010). Likewise, a high level of perception of negative dyadic coping on the part of one's partner was associated with a high level of supportive care needs both for blood cancer patients and for their partners (Weißflog et al., 2017).

In recent decades, oncological clinical studies have shown a growing interest in quality of life (Gotay et al., 1992). Although defining this concept has proved difficult (Bottomley, 2002), according to Haas (1999) “Quality of life is a multidimensional evaluation of an individual's current life circumstances in the context of the culture in which they live and the values they hold. Quality of life is primarily a subjective sense of well-being encompassing physical, psychological, social, and spiritual dimensions. In some circumstances, objective indicators may supplement or, in the case of individuals unable to subjectively perceive, serve as a proxy assessment of Quality of life.” The term health-related quality of life refers to the effects that disease and associated treatments have on the quality of life and excludes those aspects of quality of life that are not related to health (Ferrans et al., 2005). In the systematic review conducted by Bakas et al. (2012) it was pointed out that the most used models of health-related quality of life are those defined by Wilson and Cleary (1995), Ferrans et al. (2005), or World Health Organization (WHO). The model of Ferrans et al. (2005) is a revision of the model proposed by Wilson and Cleary (1995) and was chosen as the basis for this study in terms of health-related quality of life. This model includes five domains: biological, symptoms, function, general health perception, and overall health-related quality of life. Each of these areas is related to the other and there may also be reciprocal relationships. The biological field refers to the functioning of cells and various life-sustaining systems. Symptoms refer to the perception of an abnormal physical, psychological, cognitive state. Functional status considers the ability to perform tasks in various areas such as physical, social, psychological or role related. General health perception is a synthesis of health aspects, in a global assessment and the last domain of the model refers to the satisfaction of the person with the life. The model also states that these five domains are influenced by the characteristics of the person (demographic, developmental, psychological, biological) but also by the characteristics of the environment (social, physical) (Ferrans et al., 2005). In the context of a cancer diagnosis, Nayfield et al. (1992) emphasize the importance of assessing at least the following aspects of quality of life: physical, social and emotional functioning, symptoms and side effects of treatment, overall assessment of the quality of life. Because psychological suffering is often present in the case of a cancer diagnosis, emotional functioning is one of the aspects of interest in both evaluation and psycho-oncological interventions. In the present study, emotional functioning is conceptualized based on the model of Ferrans et al. (2005) as the person's perception of feeling tense, worried, nervous, irritable or sad- the emotional aspects of depression, anxiety or distress.

Since cancer is still a serious illness that impacts both the quality of life of sufferers and their partners (Kershaw et al., 2004; Tuinman et al., 2004) and the quality of their relationship (Hagedoorn et al., 2011; Ross et al., 2016), recent years have seen the appearance of research studies analyzing the relationship between dyadic coping and these aspects. These have demonstrated that common dyadic coping (Badr et al., 2010) and positive dyadic coping (Badr et al., 2018) are associated with an improvement in the functioning of the relationship and that couples' ability to act as one contributes to the quality of this (Picard et al., 2005).

Other studies have shown that common dyadic coping by partners is associated with a lower level of each member's functional quality of life (Crangle et al., 2019), while negative dyadic coping is associated with lower levels of emotional well-being in partners, as measured by Quality of Life Spouses Scale (Feldman and Broussard, 2006).

Although there are systematic reviews analyzing the quality of life in cancer patient-partner dyads (Sterba et al., 2016), their relationship quality (Kayser et al., 2018) and the link between dyadic coping and relationship quality in couples facing cancer (Traa et al., 2014), to the best of the authors' knowledge no meta-analysis that provides a quantitative analysis is available. The meta-analysis of Falconier et al. (2015) highlights relationships that are important for clinical practice by demonstrating that common dyadic coping, supportive dyadic coping and negative coping are more important predictors of relationship satisfaction than the communication of stress and delegated coping; however, it deals with a larger context than that of oncological disease. Likewise, to the best of the authors' knowledge no systematic review or meta-analysis has yet been applied to focus on the relationship between dyadic coping and the emotional functioning as part of health-related quality of life or between dyadic coping and relationship quality of the members of couples where there is a cancer diagnosis. This paper therefore intends to supply this lacuna in the literature. Its purpose is (i) to summarize the results of cross-sectional or longitudinal studies that have analyzed the relationships between dyadic coping and relationship quality and emotional functioning in couples where there is a cancer diagnosis (ii) to quantify the strength of these relationships (iii) to analyze the moderating nature of age and type of cancer on these relationships. The result of exploring the relationship between dyadic coping and the quality of the relationship and the emotional functioning can be useful information from the perspective of future interventions that by addressing dyadic coping behaviors could target results both at the intrapersonal level and at the couple level. The PRISMA guide was followed to answer these research questions.

While some studies have found that certain kinds of positive dyadic coping may be associated with a negative impact on the quality of life, possibly due to the effects of exhaustion (Crangle et al., 2019), most research associates these positive forms of dyadic coping with beneficial effects for the couple (Rottmann et al., 2015; Kayser et al., 2018). Previous studies have also brought to light the negative impacts of negative forms of dyadic coping on couples facing cancer (Feldman and Broussard, 2006; Weißflog et al., 2017). Bearing all this in mind, we would expect there to be a significant positive relationship between positive forms of dyadic coping and the relationship quality and emotional functioning of members of couples facing cancer and a significant negative relationship between negative forms of dyadic coping and their relationship quality and emotional functioning. We would expect the intensity of these relationships to depend on the type of cancer and we would also expect these relationships to be stronger in the case of older couples. We intend to carry out this analysis with regard to the communication of stress and to different forms of dyadic coping as evidenced by the STM (supportive dyadic coping by oneself/by partner, delegated dyadic coping by oneself/by the partner, common dyadic coping, negative dyadic coping by oneself/by the partner) rather than based on aggregated scores. This higher resolution identification of the relationships between the components of dyadic coping and emotional functioning and relationship quality in couples facing cancer will make it possible for future interventions to focus on those behavioral changes that have an impact on individual emotional functioning or relationship quality, depending on which of these aspects requires improvement.

## Methods

### Inclusion Criteria

To be considered eligible, studies needed to meet the following criteria.

*Criteria associated with their design:* only studies with either a cross-sectional or a longitudinal design were included in the analysis, and in the case of the longitudinal ones only the sizes of the effects that resulted from the first evaluation carried out were extracted.

*Criteria associated with the dyadic coping variable:* only studies in which dyadic coping was measured using an instrument that conceptualized it in accordance with the STM model were taken into consideration. In addition, they had to register the correlations between at least one kind of dyadic coping and relationship quality or between at least one kind of dyadic coping and emotional functioning. As well as this, only studies thatregistered these kinds of relationships for at least one of the partners were taken into account.

*Criteria associated with the relationship quality variable:* to be included in the analysis, research studies need to have measured one of the following constructs: relationship quality, quality of the marriage, relationship satisfaction, satisfaction with the marriage.

*Criteria associated with the emotional functioning variable:* to be regarded as eligible, studies needed to have used instruments for measuring the quality of life that included the emotional functioning dimension of this construct. Thus, these questionnaires had to consider the affective aspects of depression, anxiety, distress, such as sadness, worry, irritability, emotional tension.

*Criteria associated with the participants:* research papers involving participants aged at least 18 who formed couples in which one of the partners had a cancer diagnosis (regardless of the type or stage of the condition) were regarded as eligible.

### Search Strategies

Identification of relevant studies was achieved by searching the PsycINFO, PubMed and ScienceDirect online databases. Abstracts were searched using the Boolean criterion string: (cancer OR tumor OR neoplasia) AND (couple OR spouse OR partner OR dyad) AND (well-being OR wellbeing OR “quality of life” OR “relationship satisfaction”). This was done to cast the search net as widely as possible while at the same time preserving precise targeting. Only peer-reviewed English language academic journals were searched. There was no time limit on publication dates and research published up until April 2020 was considered. This search process was supplemented by a manual search of references in systematic reviews available to us on related subjects. Any articles thus identified were included in the general list which was then filtered according to the selection criteria.

### Selection Process

**Figure 1:**
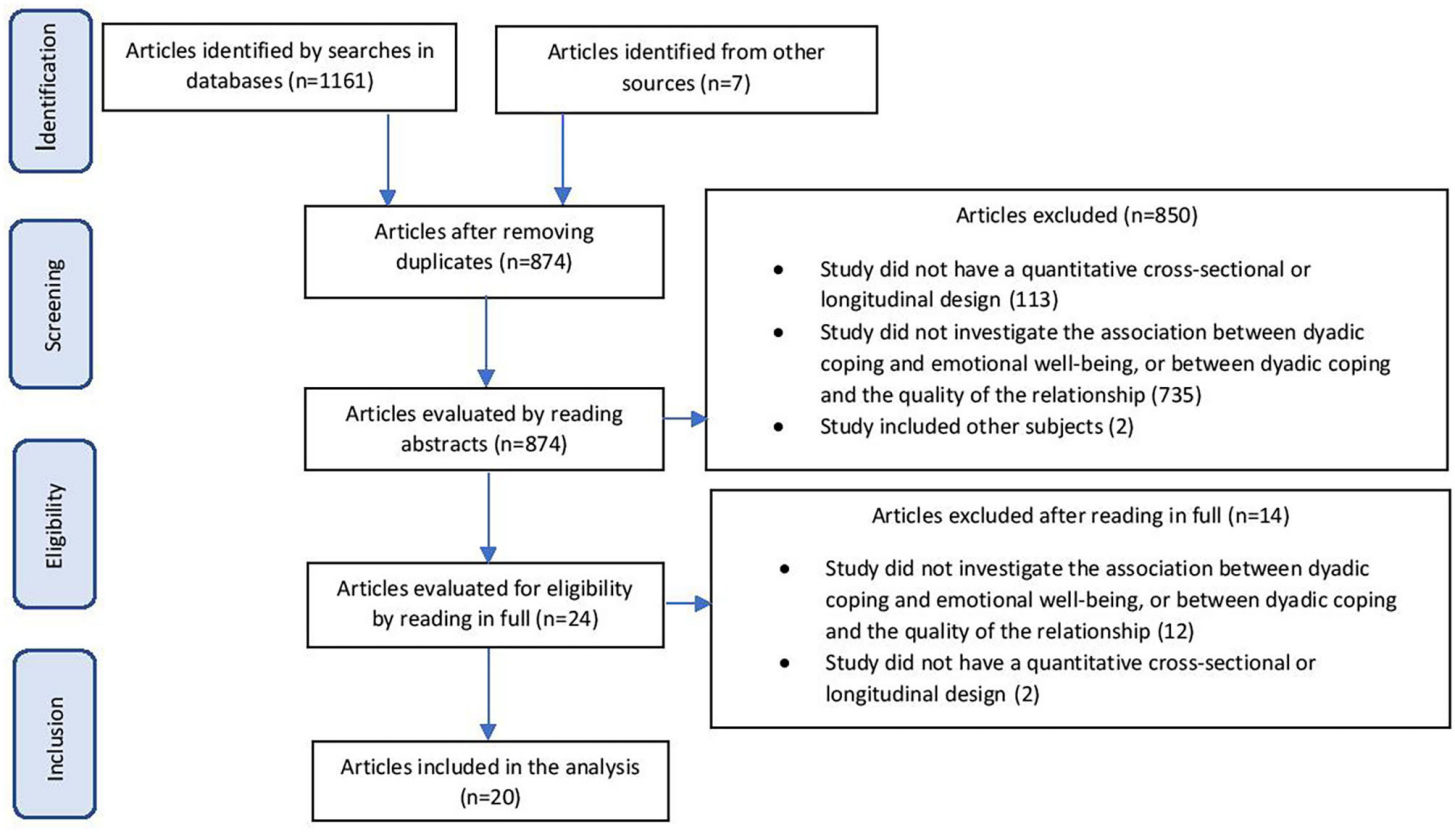
Selection process for studies.

The database search yielded 1,161 articles. Another seven studies were located following searches that used references from studies on related topics. 294 of the 1,168 were excluded as duplicates. The abstracts of the remaining 874 studies were compared with the inclusion criteria and 735 were found not to have been directed at analyzing the relationship between dyadic coping and emotional functioning and relationship quality. One hundred thirteen did not meet the design criteria and two dealt with different subjects (doctors). This left us with 24 studies to read in full and analyze. Of these, 12 had not investigated relationships of interest to our research and a further two lacked a cross-sectional or longitudinal design; 10 studies thus remained for final analysis and these formed the database for our meta-analysis. The authors worked independently on each article in the initial selection process and any differences of evaluation were resolved through discussion leading to consensus. Writers of articles who had not included data we needed for our analysis in their published papers were approached by email to furnish them. The process followed is schematized in Figure 1.

### Data Extraction

In order to prepare, administer and individually analyse the studies we devised a 27-item coding manual. These codes have been grouped into several sections: identification, sample data, design data, measured variables data, results data, effect size calculation data. The studies were divided between the first and the second author based on their appearance on the list. The coding was done independently by the first and second authors. The third author reviewed all the coding. Where there were ambiguities and potential sources of error, they were discussed to reach a consensus on the most appropriate coding decision. The data needed for the meta-analytical statistical analysis (correlation coefficient and number of participants) and information about the characteristics of participants (type of cancer, number of dyads, average age of patients, average age of partners, percentage of male patients), outcomes measures, the nature of the studies (cross-sectional or longitudinal) and their principal results were extracted from the articles.

### Meta-Analytical Strategy

Statistical analysis was performed using the Comprehensive Meta-Analysis software v3.3 (Borenstein et al., 2013). The size of effect used for this meta-analysis was the Bravis-Pearson *r* correlation coefficient. This type of analysis (Borenstein et al., 2009) transforms the *r* coefficient into Fisher's *z* for the processing of the meta-analytical calculation but at the end the result is converted back into the *r* correlation coefficient. The purpose of these transformations is that when Fisher's *z* is used the dispersion associated with each measure of size of effect depends exclusively on the sample size whereas if the *r* is used directly the dispersion related to size of effect is dependent both on sample size and on the size of the correlation coefficient. The *r* coefficient was calculated for relationships between kinds of dyadic coping and emotional functioning and between kinds of dyadic coping and relationship quality both for patients and for their life partners. Because the studies analyzed differed both in terms of the characteristics of participants (patients had been diagnosed with different types of cancers and had been recruited in different medical centers) and in terms of methodology (different evaluative instruments having been applied), we assumed the existence of random variation in the true size of the effect from one study to another and therefore applied a meta-analysis that assumes a random-effect. This method of analysis also permits a greater degree of generalization than the fixed effects model (Hedges and Vevea, 1998). To investigate the moderating nature of the age and the type of cancer, meta-regressions were applied as recommended by Borenstein et al. (2015). While in empirical studies the sample size is equal to the number of participants (*N*), in meta-analysis the sample size is given by the number of studies included (*k*). The *I*^2^ index (Higgins et al., 2003) was used to estimate inter-study heterogeneity. This statistic corresponding to the percentage of observed dispersion due to real differences, as distinct from random variations, between the values of the measures of size when comparing studies. It can take values ranging from 0 to 100% with a value of 75% typically being regarded as high, 50% being described as “moderate” and 25% as “low,” according to the initial suggestion of those who first devised it.

### Sources of Bias

Since any such systematic review may be affected by publication bias this aspect was analyzed by calculation of Egger's intercept (Egger et al., 1997). Additionally, to evaluate the studies included we used the STROBE (STrengthening the Reporting of OBservational studies in Epidemiology) checklist for observational studies (von Elm et al., 2007).

## Results of the Systematic Review

A summary of the characteristics of the studies is presented in Table 1.

**Table 1:** Results of the systematic literature review (*k* = 10).

**Study ID**	**Dyadic aim**	**Design**	**Study population** **(type of cancer,** **no of diads,** **% male patients, average** **age patients, average** ** age partners)**	**Measures**		**Main conclusions**	**Risk of bias**
				**Patients**	**Partners**		
Acquati and Kayser (2019) (USA)	The impact of illness on the QoL and dyadic coping, the influence of relational mutuality on dyads' coping in case of younger and middle-aged couples	Cross-sectional	*Cancer:* breast *No dyads:*86 *% male patients:* 0 *Average age patients:* 46.6 *Average age partners:* 49.1	*DC*: Dyadic Coping Scale *QoL*: Functional Assessment of Cancer Therapy- Breast (FACT-B) *Relational mutuality*: Mutual Psychological Development Questionnaire (MPDQ)	*DC*: Dyadic Coping Scale *QoL*: Quality of Life Questionnaire for Spouses (QL-SP), Illness Intrusiveness Ratings Scale (IIRS) *Relational mutuality*: Mutual Psychological Development Questionnaire (MPDQ)	Younger couples reported statistically significant worse QoL and dyadic coping scores than the middle-age couples. For younger dyads, coping styles (positive and negative) were the result of both actor and partner effects of mutuality	Low
Badr et al. (2010) (USA)	Prospective evaluation of association between dyadic coping and cancer-related distress and dyadic adjustment in couples facing metastatic breast cancer	Longitu dinal	*Cancer:* breast *No dyads:*191 *% male patients:*0 *Average age patients:* NS *Average age partners:* NS	*DC*: Dyadic Coping Questionnaire (FDCT-N) *Distress*: Impact of Event Scale (IES) *RQ*: Dyadic Adjustment Scale (DAS-7)	*DC*: Dyadic Coping Questionnaire (FDCT-N) *Distress*: Impact of Event Scale (IES) *RQ*: Dyadic Adjustment Scale (DAS-7)	More common positive dyadic coping and less common negative dyadic coping was associated with greater dyadic adjustment for patients and partners Effects of common positive dyadic coping on cancer-related distress significantly differed for patients and their partners (partners reported lower levels of distress, patients reported higher levels of distress) Common negative dyadic coping was always significantly associated with distress and the relation was stronger for patients	Low
Badr et al. (2018) (USA)	Relations between patients' and spouses' dyadic coping and their own/each other's psychological and marital adjustment. Associations between changes in dyadic coping and changes in patients' and spouses' psychological and marital adjustment	Cross-sectional (secondary analysis of a randomized pilot trial)	*Cancer:* head and neck *No dyads:*60 *% male patients:* 30 *Average age patients:* 58.43 *Average age partners:* 58.07	*DC*: Dyadic Coping Inventory (DCI) *Anxiety, depression*: Patient-Reported Outcomes Measurement Information System (PROMIS) *RQ*: Dyadic Adjustment Scale (DAS-7)	*DC*: Dyadic Coping Inventory (DCI) *Anxiety, depression*: Patient-Reported Outcomes Measurement Information System (PROMIS) *RQ*: Dyadic Adjustment Scale (DAS-7)	Significant actor effects were found for problem-focused stress communication, problem-focused dyadic coping, emotion-focused dyadic coping on marital adjustment. Actor and partner effects for negative dyadic coping were also significant. Also, significant actor effects of problem-focused stress communication and problem-focused dyadic coping were noticed on depression	Low
Crangle et al. (2019) (Canada)	Whether common dyadic coping mediates the associations between attachment and quality of life	Cross-sectional	*Cancer:* ovarian *No dyads:*106 *% male patients:* 0 *Average age patients:* 59.1 *Average age partners:* 60.8	*DC:* Dyadic Coping Inventory (DCI) *Adult attachment*: Close Relationships Scale—Revised (ECR-R) *QoL*: Functional Assessment of Cancer Therapy (FACT)-Ovarian	*DC*: Dyadic Coping Inventory (DCI) *Adult attachment*: Close Relationships Scale—Revised (ECR-R) *QoL*: Functional Assessment of Cancer Therapy (FACT)-general population	Worse social and functional QOL were associated with one's own and one's partner's greater insecure attachment and this relation was mediated by common dyadic coping. Greater common dyadic coping reported by one's partner was associated with one's own lower functional QOL	Low
Ernst et al. (2017) (Germany)	The impact of dyadic coping on QoL	longitudinal	*Cancer:* hematologic *No dyads:*208 *% male patients:* 62 *Average age patients:* 57.7 *Average age partners:* 56.9	*DC:* Dyadic Coping Inventory (DCI) *QoL*: SF-12 Health Survey	*DC:* Dyadic Coping Inventory (DCI) *QoL*: SF-12 Health Survey	DC (t1) had a partner effect on physical QoL (t2) and an actor and partner effect on mental QoL(t2) Different subtypes of DC had actor and partner impact on patient's or partner's QoL	Low
Feldman and Broussard (2006) (USA)	Men's dyadic coping when their partners are diagnosed with breast cancer	Cross-sectional	*Cancer:* breast *No dyads:* 0 (71 partners) *% male patients:* NA *Average age patients:* NA *Average age partners:* 51	-	*DC*: Dyadic Coping Scale (DCS) *QoL*: Quality of Life Spouses Scale (QOL-SP) *Illness intrusiveness*: Illness Intrusiveness Rating Scale (IIRS)	Significant associations were noticed between dyadic coping styles and illness intrusiveness	Low
Pankrath et al. (2018) (Germany)	How the relationship satisfaction is affected by the dyadic coping	Cross-sectional	*Cancer:* haematologic *No dyads:* 327 *% male patients:* 63.3 *Average age patients:* 57 *Average age partners:* 56	*DC:* Dyadic Coping Inventory (DCI) *RQ*: Partnership Questionnaire (PFB-K) *Anxiety, depression*: PHQ-4	*DC:* Dyadic Coping Inventory (DCI) *RQ*: Partnership Questionnaire (PFB-K) *Anxiety, depression*: PHQ-4	A significant positive association was noticed between positive DC and relationship satisfaction while negative DC was related to lower levels of relationship satisfaction. Age, distress and duration of relationship duration had moderating effects on the association between DC and relationship satisfaction A negative significant association was highlighted between partners' distress and the relationship satisfaction of the partners	Low
Regan et al. (2014) (Australia)	Dyadic coping affects patients 'and their wives' anxiety, depression and relationship satisfaction differently (wives are more likely than patients to be influenced by their own and their partner's dyadic coping)	Cross-sectional	*Cancer:* prostate *No dyads:* 42 *% male patients:* 100 *Average age patients:* 63.7 *Average age partners:* 59.6	*DC:* Dyadic Coping Inventory (DCI) *RQ*: Revised-Dyadic Adjustment Scale (R-DAS) *Anxiety, depression*: Hospital Anxiety and Depression Scale (HADS)	*DC:* Dyadic Coping Inventory (DCI) *RQ*: Revised-Dyadic Adjustment Scale (R-DAS) *Anxiety, depression*: Hospital Anxiety and Depression Scale (HADS)	A significant association was highlighted between relationship satisfaction and patients' and wives' positive and negative dyadic coping, and same strategies of their partners'. Partner's use of supportive dyadic coping was related with anxiety and depression. Husbands' and wives' perceptions of their partner's negative dyadic coping was also related with anxiety and depression	
Rottmann et al. (2015) (Denmark)	The relationship over time between different forms of dyadic coping and relationship quality and depressive symptoms	longitudinal	*Cancer:* breast *No dyads:* 538 *% male patients:* 0 *Average age patients:* 58 *Average age partners:* 60.1	*DC:* Dyadic Coping Inventory (DCI) *RQ*: ladder with steps numbered 0 through 10, where 0 represents the worst possible, and 10 the best possible, relationship *Depression*: Center for Epidemiologic Studies- Depression Scale (CES-D)	*DC:* Dyadic Coping Inventory (DCI) *RQ*: ladder with steps numbered 0 through 10, where 0 represents the worst possible, and 10 the best possible, relationship *Depression*: Center for Epidemiologic Studies- Depression Scale (CES-D)	All participants experienced more depressive symptoms the more delegated coping the patients provided to the partners A negative association was noticed between the delegated coping offered by the partners to the patients and their depressive symptoms The common dyadic coping was positive associated with relationship quality and was negative associated with depressive symptoms of patients and partners The negative dyadic coping was inverse associated with patients' and partners' outcomes	Low
Zimmermann et al. (2010) (Germany)	Individual factors, dyadic variables and individual variables of man as predictors of body image in women with breast cancer	Cross-sectional	*Cancer:* breast *No dyads:* 98 *% male patients:* 0 *Average age patients:* 51.9 *Average age partners:* 53.1	*DC*: Dyadic Coping Questionnaire *RQ*: Quality of Marriage Index (QMI), Abbreviated Dyadic Adjustment Scale (ADAS) *Depression*: Hospital Anxiety and Depression Scale (HADS) *Body image*: Self Image Scale (SIS)	*DC*: Dyadic Coping Questionnaire *RQ*: Quality of Marriage Index (QMI), Abbreviated Dyadic Adjustment Scale (ADAS) *Depression*: Hospital Anxiety and Depression Scale	Women's self-acceptance was predicted by women's depressive symptoms and men's marital satisfaction Women's perceptions of their partner's acceptance of their appearance was predicted by relationship satisfaction and perspective on common dyadic coping	Low

*DC, Dyadic Coping; QoL, Quality of Life; RQ, relationship quality; NA, Not Applicable, NS, Not Specified*.

## Meta-Analysis Results

### The Association Between Common Dyadic Coping and Relationship Quality

**Figure 2:**
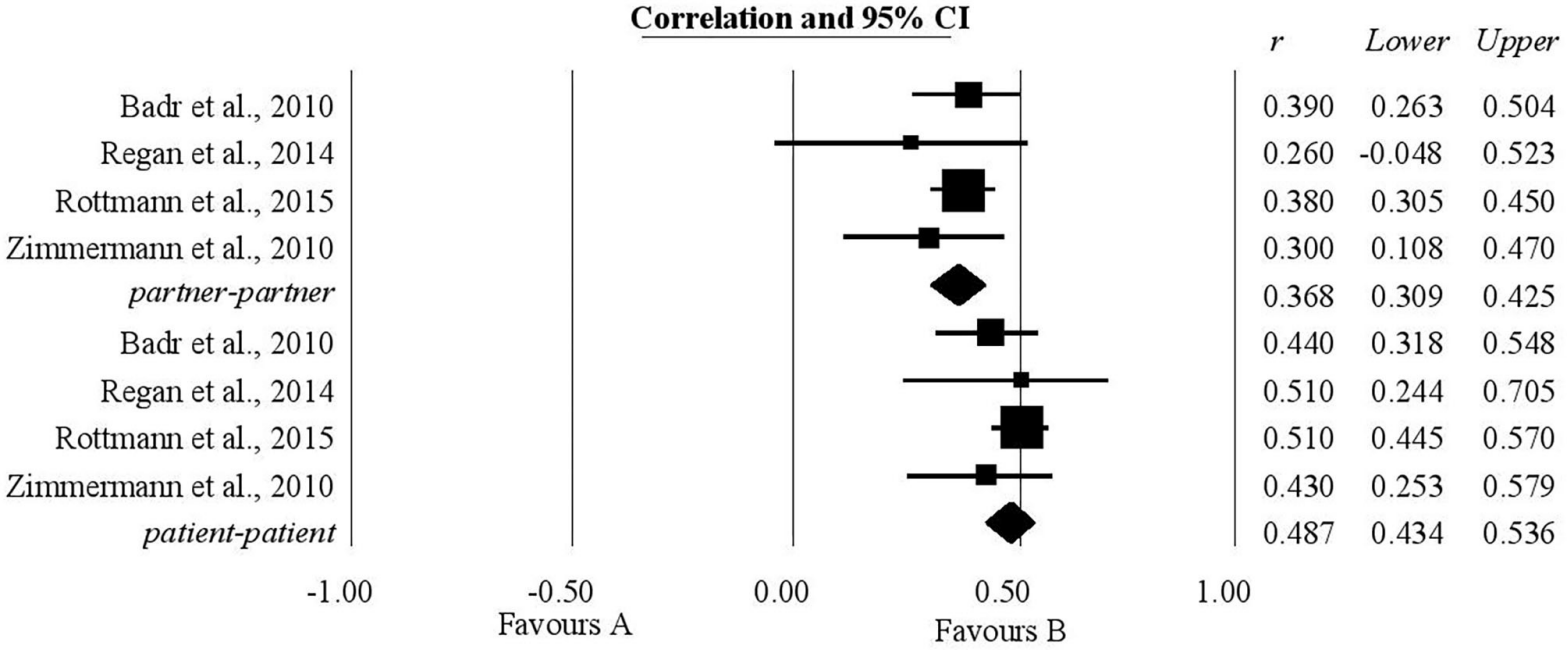
Associations reported between common dyadic coping and relationship quality.

The authors' expectations regarding the relationship between common dyadic coping and relationship quality were borne out both as concerning patients and as concerning their partners. For patients, the analysis included four studies and the coefficient of correlation, *r*, was statistically significant, having a value of 0.48, with the confidence interval (0.43, 0.53). For life partners, the analysis again included four studies and the coefficient of correlation obtained was statistically significant, having a value of 0.36 (0.30, 0.42) (Figure 2).

### The Association Between Common Dyadic Coping and Emotional Functioning

**Figure 3:**
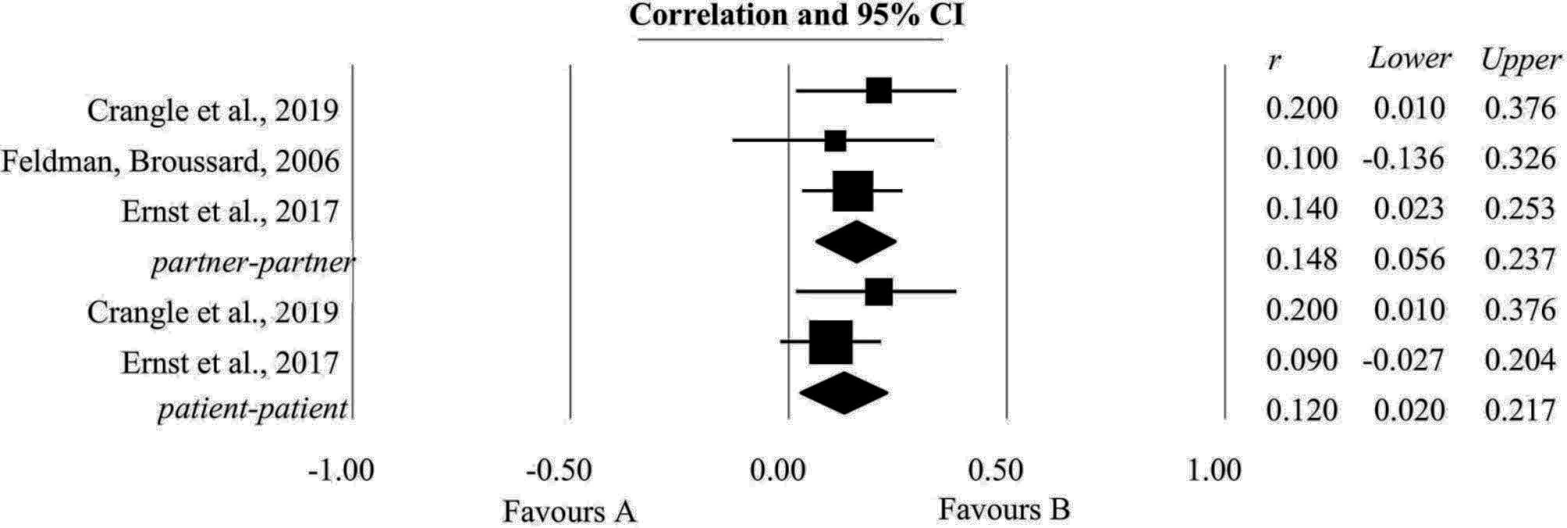
Association reported between common dyadic coping and emotional functioning.

A statistically significant positive association was found between common dyadic coping and emotional functioning both for patients and for their partners (Figure 3). Two studies provided information about this association for patients; the coefficient of correlation was 0.12 with a confidence interval of (0.02, 0.21). For their partners, information extracted from three studies was analyzed, giving a coefficient of correlation r with a value of 0.14 (0.05, 0.23).

### The Association Between Communication of Stress by Oneself and Relationship Quality

**Figure 4:**
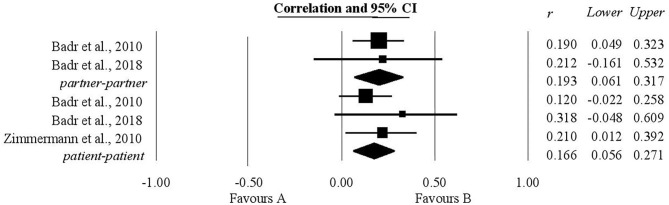
Associations reported between the communication of stress by oneself and relationship quality.

A statistically significant positive relationship was also found between the communication of stress by oneself and relationship quality. Three studies provided information regarding patients and two gave information about this relationship in the case of their partners. The coefficient of correlation obtained for patients was 0.16 (0.05, 0.27). The coefficient of correlation obtained for partners was 0.19 with a confidence interval of (0.06, 0.31). These results are shown in Figure 4.

### The Association Between Supportive Dyadic Coping by Oneself and Relationship Quality

**Figure 5:**
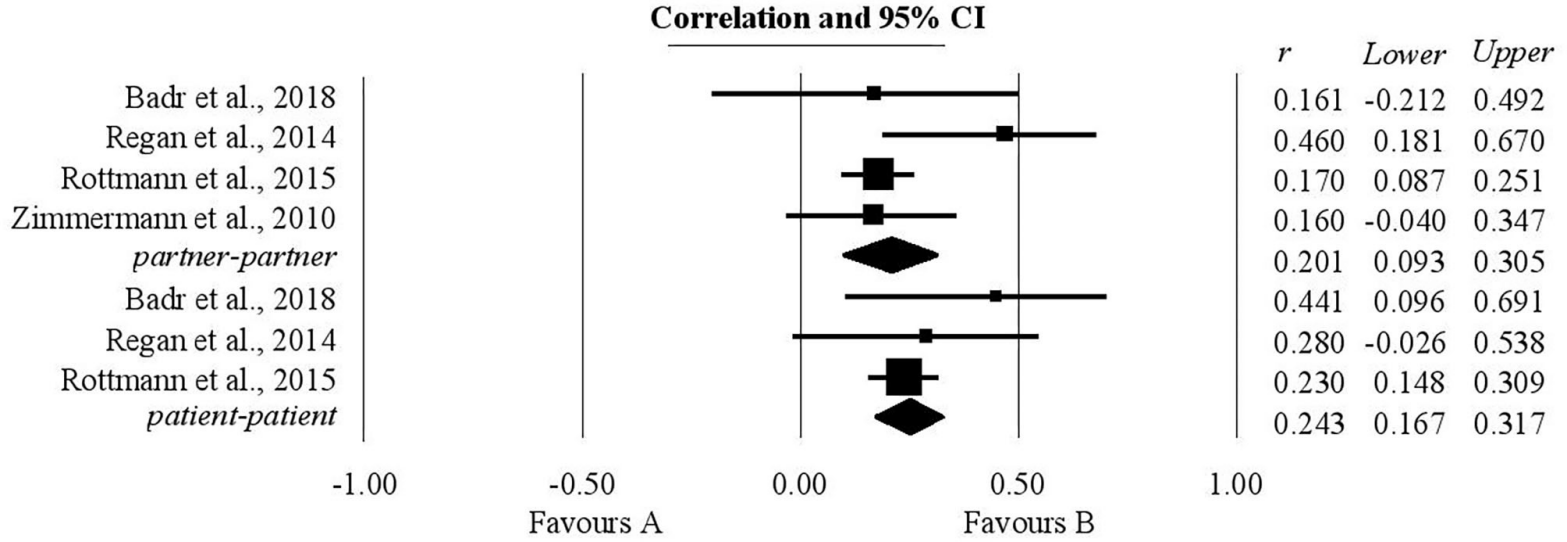
Associations reported between supportive dyadic coping by oneself and relationship quality.

The authors' expectations regarding the relationship between supportive dyadic coping by oneself and relationship quality were borne out both for patients and for their partners. For patients, three studies were analyzed and the coefficient of correlation r, statistically significant, had a value of 0.24 (0.16, 0.31). For their life partners, four studies were analyzed, giving a statistically significant correlation of 0.2 lying within a confidence interval (0.09, 0.3) (Figure 5).

### The Association Between Supportive Dyadic Coping by Partner and Relationship Quality

**Figure 6:**
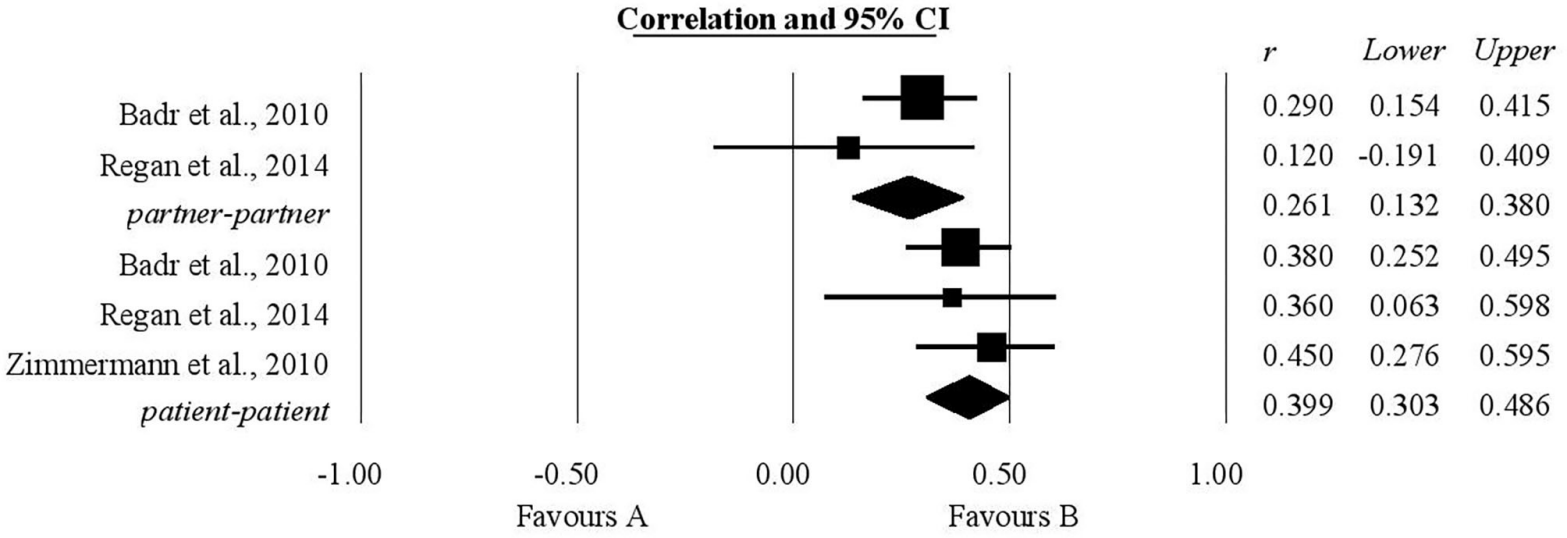
Associations reported between supportive dyadic coping by partner and relationship quality.

Both for patients and for their partners the relationship between supportive dyadic coping by partner and relationship quality was positive and statistically significant (Figure 6). For patients the coefficient of correlation obtained was 0.39 (0.3, 0.48), while for their partners the correlation coefficient was 0.26 within the confidence interval (0.13, 0.38). For patients the analysis included three studies and for their partners two studies.

### The Association Between Negative Dyadic Coping by Oneself and Relationship Quality

**Figure 7:**
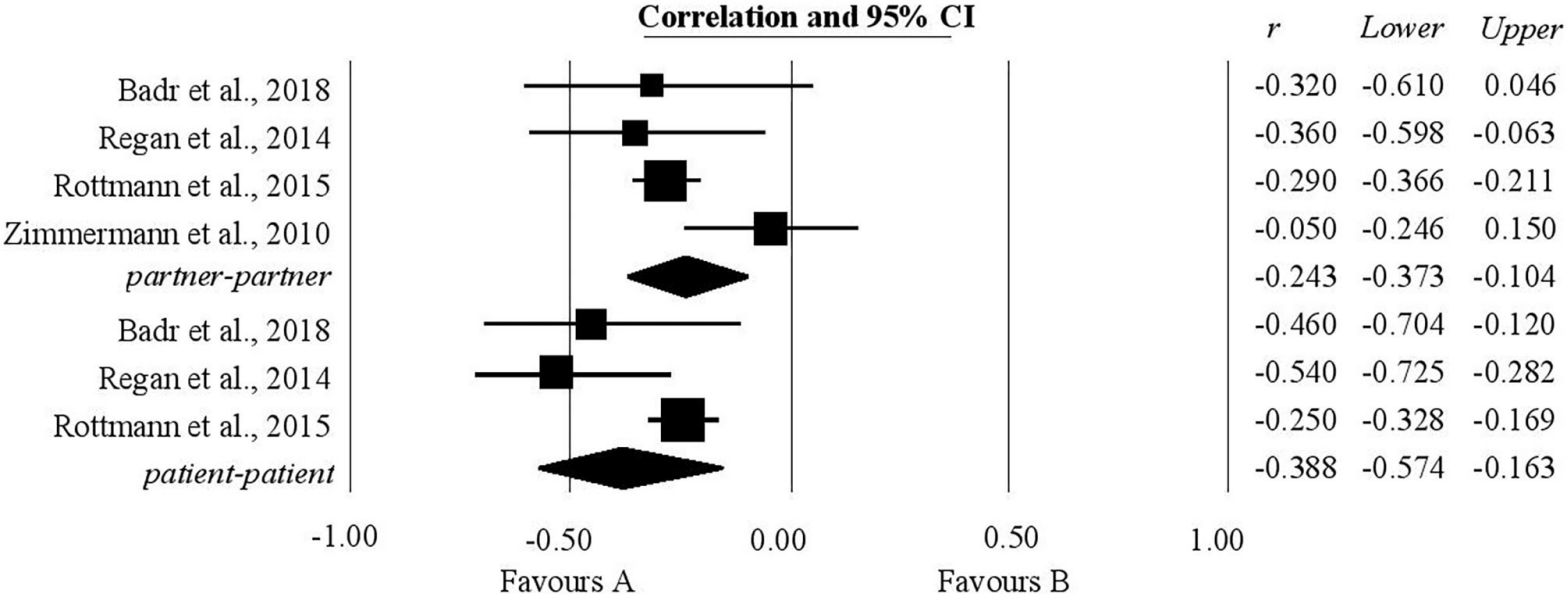
Associations reported between negative dyadic coping by oneself and relationship quality.

As expected, the analysis showed a statistically significant negative relationship between negative dyadic coping by oneself and relationship quality for all participants. For patients, three studies were analyzed with respect to this relationship and a correlation of −0.38 (−0.57, −0.16) was calculated. For partners, the analysis included four studies and the coefficient of correlation had a value of −0.24 within the confidence interval (−0.37, −0.1) (Figure 7).

### The Association Between Negative Dyadic Coping by Partner and Relationship Quality

**Figure 8:**
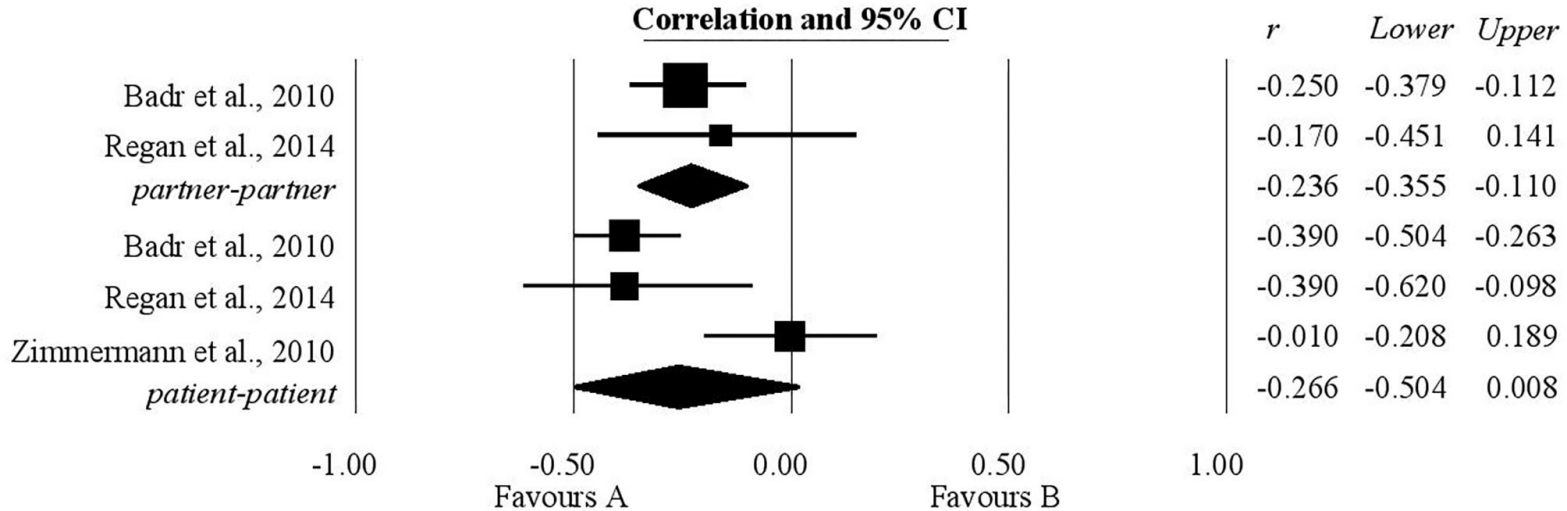
Associations reported between negative dyadic coping by partner and relationship quality.

For the relationship between negative dyadic coping by partner and relationship quality, our expectations were only partially confirmed. It was only for partners, after an analysis of the two studies that provided data on this point, that a statistically significant negative correlation was found, the value being −0.23 within the confidence interval (−0.35, −0.11) (Figure 8).

Table 2 provides a summary of the results.

**Table 2:** Effect sizes of outcomes.

**Evaluated relationship**	**No. of studies**	**No. of participants**	**Correlation (95% CI)**	***Q***	***I^**2**^***	**Egger's t test for publication bias**
	***K***	***N***				
**Common dyadic coping- emotional functioning**						
Patients-Patients	2	386	0.12 (0.02 to 0.21)^*^	0.95	0.00	FS
Partners-Partners	3	457	0.14 (0.05 to 0.23)^**^	0.47	0.00	0.04
All participants	3	843	0.13 (0.06 to 0.20)^**^	1.24	0.00	0.43
**Common dyadic coping- relationship quality**						
Patients-Patients	4	869	0.48 (0.43 to 0.53)^**^	1.70	0.00	−0.79
Partners-Partners	4	869	0.36 (0.30 to 0.42)^**^	1.34	0.00	−1.13
All participants	4	1,738	0.42 (0.39 to 0.46)^**^	1.84	0.00	−1.36
**Stress communication by oneself- relationship quality**						
Patients-Patients	3	319	0.16 (0.05 to 0.27)^**^	1.32	0.00	1.86
Partners-Partners	2	221	0.19 (0.06 to 0.31)^**^	0.01	0.00	FS
All participants	3	540	0.17 (0.09 to 0.25)^**^	0.77	0.00	1.28
**Supportive dyadic coping by oneself- relationship quality**						
Patients-Patients	3	610	0.24 (0.16 to 0.31)^**^	1.53	0.00	1.07
Partners-Partners	4	708	0.20 (0.09 to 0.30)^**^	3.96	24.23	0.97
All participants	4	1,318	0.22 (0.15 to 0.29)^**^	3.45	13.14	0.99
**Supportive dyadic coping by partner- relationship quality**						
Patients-Patients	3	331	0.39 (0.30 to 0.48)^**^	0.54	0.00	0.08
Partners-Partners	2	233	0.26 (0.13 to 0.38)^**^	1.02	2.26	FS
All participants	3	564	0.34 (0.25 to 0.43)^**^	2.48	19.5	0.22
**Negative dyadic coping by oneself- relationship quality**						
Patients-Patients	3	610	−0.38 (−0.57 to −0.16)^**^	5.64	64.59	−2.25
Partners-Partners	4	708	−0.24 (−0.37 to −0.10)^**^	5.58	46.24	0.43
All participants	4	1,318	−0.28 (−0.42 to −0.13)^**^	9.46	68.3	−0.42
**Negative dyadic coping by partner- relationship quality**						
Patients-Patients	3	331	−0.26 (−0.50 to 0.008)	10.81	81.5	–
Partners-Partners	2	233	−0.23 (−0.35 to −0.11)^**^	0.22	0.00	FS
All participants	3	564	−0.21 (−0.39 to −0.02)^*^	7.97^*^	74.93	3.13

* *P <0.05*;

** *P <0.001; FS, Few Studies*.

### Additional Analysis

In addition to the analyzes that we initially intended to perform, supplementary analyzes were included that consider the associations of interest at the level of all participants, not only at the level of subgroups formed by patients and their partners. All results obtained were statistically significant. Where possible, the results obtained were compared with those highlighted in the meta-analysis performed by Falconier et al. (2015). Thus, it was found that for the associations between negative dyadic coping by partner, stress communication by self, supportive dyadic coping by partner, supportive dyadic coping by self, common dyadic coping and satisfaction in the relationship, the confidence intervals do not overlap. Because these statistics have non-overlapping confidence intervals, they are significantly different. Since the confidence intervals associated with the correlation coefficients obtained in the two meta-analyzes for the association between negative dyadic coping by self and relationship quality overlap, we cannot say with certainty that these statistics are significantly different. Therefore, statistical tests were performed to clarify this issue. In order to test the hypothesis that there is zero correlation between the correlation coefficients obtained in these two studies, the RStudio software version 1.2.5042 was used. The results obtained pointed out that there is no correlation between the correlation coefficients obtained in the meta-analysis conducted by Falconier et al. (2015) and in the present meta-analysis regarding the association between negative dyadic coping by the partner, negative dyadic coping by self, stress communication by self, supportive dyadic coping by the partner, supportive dyadic coping by self, common dyadic coping and the relationship quality.

Table 3 shows the dimensions of dyadic coping for which the associations with the relationship quality could be compared between the two meta-analyzes.

**Table 3:** Comparison of the results obtained in the two meta-analyzes.

**Dyadic coping dimension**	**Summary information Falconier et al**.		**Summary information current study**		**Test statistic *z***	**2-tail *p***
	***k***	***r* (95% CI)**	***k***	***r* (95% CI)**		
Negative dyadic coping by partner	24	−0.48 (−0.53, −0.43)^**^	3	−0.21 (−0.39 to −0.02)^*^	0	1
Negative dyadic coping by self	30	−0.37 (−0.42, −0.33)^**^	4	−0.28 (−0.42 to −0.13)^**^	−0.1	0.92
Stress communication by self	20	0.34 (0.29, 0.39)^**^	3	0.17 (0.09 to 0.25)^**^	0	1
Supportive dyadic coping by partner	32	0.57 (0.50, 0.63)^**^	3	0.34 (0.25 to 0.43)^**^	0	1
Supportive dyadic coping by self	34	0.39 (0.34, 0.45)^**^	4	0.22 (0.15 to 0.29)^**^	0.18	0.85
Common dyadic coping	30	0.53 (0.48, 0.57)^**^	4	0.42 (0.39 to 0.46)^**^	0.14	0.89

* *P <0.05*;

** *P <0.001*.

### Moderator Analysis

Attempts were made to apply meta-regressions to investigate the moderating nature of age and cancer type, but the small number of studies did not allow the analysis. Meta-regression could be applied only to study the moderating character of the age in terms of the relationship between the supportive dyadic coping by oneself and the relationship quality. The result obtained was statistically insignificant.

### The Risk of Bias

The risk of bias in individual studies was assessed based on the STROBE checklist (STrengthening the Reporting of OBservational studies in Epidemiology) checklist for observational studies (von Elm et al., 2007). Thus, for each study, each item of this checklist was considered. The scoring was done as follows: if the study considered the aspect described by that item it was marked with “0,” otherwise it was marked with “1.” For items that required checking several aspects, the value “1” was divided according to the number of targeted aspects. The final score corresponding to the risk of bias for each study was obtained by summing the scores obtained for each item. Following this approach, it was found that the studies included in the analysis have a low risk of bias with scores corresponding to the risk ranging between 1.4 and 4. The less treated issues referred to how the sample size was calculated, which were the ways to address possible sources of bias, how the missing data were approached.

## Discussion

To the authors' knowledge this is the first meta-analysis to study the relationship between dyadic coping conceptualized according to the STM model and both relationship quality and emotional functioning in couples facing a cancer diagnosis. To this end we analyzed ten articles identified using systematic searches in the PsycINFO, PubMed and ScienceDirect databases. This piece of research has several strengths, including the use of strict inclusion/exclusion criteria and the application of meta-analytical techniques of processing data which facilitated the bringing together of results from different studies, but it also has the weakness that only a small number of studies were included in the final analysis.

The meta-analyses carried out largely confirmed our expectations regarding the relationships between dyadic coping and both relationship quality and emotional functioning in couples in which one member has been diagnosed with cancer. Statistically significant positive associations were demonstrated between common dyadic coping, the communication of stress by oneself, supportive dyadic coping by oneself/by partner and relationship quality both for cancer patients and for their life partners. It was also shown that there is a statistically significant negative correlation between negative dyadic coping by oneself and relationship quality for both members of the couple and a statistically significant negative correlation between negative dyadic coping by partner and relationship quality, but only for non-patient partners. Additionally, a statistically significant positive relationship was also found between shared dyadic coping and emotional functioning for both patients and their partners.

The strongest effects were found in terms of the relationship between common dyadic coping and the quality of the relationship for both patients and partners. However, although weaker, statistically significant effects were also present in the relationship between common dyadic coping and the emotional functioning of patients and their partners. These results suggest that this dyadic process is important in couples facing cancer not only in terms of couple-level outcomes but also in terms of individual-level outcomes. Also, both the perception of one's own coping and the perception of one's partner's coping is significantly associated with the quality of the relationship, which emphasizes the importance of both partners' behaviors for relationship satisfaction. Another aspect worth emphasizing is that both positive and negative forms of dyadic coping have been significantly associated with the quality of the relationship, but in opposite directions, an aspect that may be important in the design of future interventions. These results can be explained by the fact that the forms of positive dyadic coping include taking over the partner's tasks to help him but also finding solutions together and empathic support, strategies that can help strengthen couple cohesion thus facilitating improving the relationship quality. Carrying out pleasant activities together with the purpose of relaxation leads to the reduction of stress and can also contribute in this way to an increase in the relationship quality. Communication on various stressful aspects of the disease can also contribute to improving the relationship quality by lowering the level of stress. Besides, engaging in strategies specific to positive dyadic coping can lead to increased trust that the two partners have in each other and to enhance the feeling of belonging. On the other hand, the negative forms of dyadic coping imply that the support provided is accompanied by a lack of empathy, which can reduce openness and intimacy, thus affecting the relationship quality. The relationship quality can be negatively impacted by the presence of disinterest, of distance that can affect the feeling of belonging. Minimizing the partner's stress can lead to the fact that the relationship is not seen as a source of support in difficult circumstances diminishing the level of trust, increasing the perceived stress, and thus negatively influencing the relationship quality.

In most cases, the small number of studies did not allow the analysis of the moderating nature of the age and type of cancer. Meta-regression could be applied only to study the moderating character of age in terms of the relationship between the supportive dyadic coping by oneself and the relationship quality and the result obtained was statistically insignificant.

The results of the meta-analysis demonstrate the importance of the communication of stress and of different forms of dyadic coping for the relationship quality and emotional functioning of couples facing cancer. The significant positive connection between the communication of stress and relationship quality may be explained by the fact that this kind of communication can achieve a better match between felt needs and support received (Cutrona and Russell, 1990). The significant positive connection between supportive dyadic coping and relationship quality may also be understood through the fact that, in the context of the disease, what the partners need to do in following treatment and in day-to-day life can be challenging, with the result that resolving of problems and the giving and receiving of support in achieving concrete tasks can be particularly important and can lead to an increase in cohesion between the couple. Common dyadic coping too is significantly positively associated with relationship quality, possibly because a coordinated and shared approach to the disease improves the feeling of closeness in the relationship (Kayser et al., 2007). This coordinated response to the disease can facilitate the employment of appropriate coping strategies capable of having a positive effect on psychological adjustment to the disease both in patients and in their partners (Manne et al., 2004), which contributes to the positive connection between common dyadic coping and the emotional functioning of the members of the couple. At the same time negative dyadic coping was associated with lower relationship quality, which may be understood in the light of the fact that this type of coping does not show an attitude of respect toward the partner, one which appreciates their resourcefulness, but rather displays disinterest and a minimizing of the problems they are facing.

The results of the present analysis are in harmony with those obtained by Falconier et al. (2015) in their meta-analysis, namely that both positive and negative dyadic coping make a significant contribution to couple relationship quality, but in opposite directions; however, that meta-analysis deals with a wider context than that of stress caused by cancer and considers several models of dyadic coping not only STM. Thus, only two of the studies included in the meta-analysis performed by Falconier et al. (2015) met the eligibility criteria of this research and are found in the present analysis (Badr et al., 2010; Zimmermann et al., 2010). If we refer to the magnitudes of the effects obtained for the relationships studied by both meta-analyzes: the association between supportive dyadic coping by oneself / by the partner, communication of stress by oneself, negative dyadic coping by oneself / by partner and relationship quality, we notice that those obtained in the present meta-analysis are inferior to those obtained by Falconier et al. (2015). The closest values in terms of effect size were obtained in these studies for the associations between the common dyadic coping and the quality of the relationship. It should be noted that for the correlations obtained in the present meta-analysis between the common dyadic coping of patients, partners and all participants and the quality of the relationship, the confidence intervals are relatively narrow which leads to high confidence in point estimates. For the other correlation coefficients calculated in this study, the confidence interval is wider which leads to greater uncertainty regarding the effect size. Also, the statistical tests performed showed that there is no association between the correlation coefficients determined in the present meta-analysis and the meta-analysis performed by Falconier et al. (2015) for the associations between supportive dyadic coping by oneself/by the partner, communication of stress by oneself, negative dyadic coping by oneself/by the partner, common dyadic coping and relationship quality. This fact highlights that the correlations between the mentioned components of dyadic coping and the relationship quality are different in the context of cancer compared to the broader context of different stressors considered by Falconier et al. (2015). In the oncological context, these associations are weaker, which raises the question of whether they are influenced by other psychological variables and what they would be, whether these variables are individual or dyadic and whether they refer to characteristics of the disease or treatments. Thus, future studies could investigate for example the possible influence of the prevalence of physical or mental symptoms, disease characteristics, body image.

When talking about dyadic coping in the context of cancer, our results are in line with those reported by Traa et al. (2014) in their systematic review, which showed that resolving problems together, supportive behaviors, positive dyadic coping and open, constructive communication about the disease are associated with better functioning of the relationship than when dysfunctional communication patterns, unsupportive behaviors and negative dyadic coping are adopted. These were associated with less functional relationships. In the context created by renal transplant, the dyadic coping of male patients was positively associated with their own satisfaction in the relationship and also with their female partners' satisfaction in the relationship, but the dyadic coping of their female partners was positively associated only with their own satisfaction in their relationship and not also with the satisfaction of the male patients (Tkachenko et al., 2019).

Regarding the relationship between common dyadic coping the emotional functioning of patients, this has not previously been examined in any reviews of the literature. The review of Sterba et al. (2016) did investigate the quality of life for dyads formed of patients with a diagnosis of cancer of the head or neck and their partners and drew attention to the fact that psychological quality of life had been the most studied construct, but results had varied between studies, possibly because of differences in the research questions and variability in the participants. In regard to chronic obstructive pulmonary disease it was found that a more sustained practice of negative dyadic coping and a lower degree of positive dyadic coping was associated with a lower quality of life and a higher level of distress (Meier et al., 2011), and that the greater the disparity between the levels of perceived delegated coping for each couple, the poorer their quality of life. It was likewise apparent that in the case of partners, quality of life was influenced by the communication of stress by patients and also by their negative dyadic coping, as measured at an earlier point (Vaske et al., 2015).

Besides relationships of interest for the present study, the research papers included in the present analysis also report other results that are significant in the context of dyad-centered psycho-oncological research. These include the fact that for younger couples mutuality in the relationship influences dyadic coping at both a personal and an interpersonal level (Acquati and Kayser, 2019), that for women facing cancer in metastasis and their partners common dyadic coping influences partners' level of distress due to the disease differently (Badr et al., 2010), and that relationship satisfaction and their perspective on common dyadic coping is an accurate predictor of the perception of patients facing breast cancer regarding their partners' acceptance of their appearance (Zimmermann et al., 2010).

Although the results of the meta-analyses we performed largely confirmed our expectations and were also in harmony with those of previous studies conducted in the context of cancer but also in the wider one of other conditions, they need to be interpreted with caution given the small number of studies analyzed. This small number of studies is a limitation of this research and arises for various reasons. The first of these to acknowledge is our strict inclusion criteria. This policy contributed to a clear delimitation of our area of interest, to the use in the studies analyzed of a single clear concept of dyadic coping, and to an easier application of the meta-analytical techniques, but resulted in a reduction in the number of eligible studies. Another possible explanation is that the development of psycho-oncological research has only relatively recently widened its attention from the individual (patient or partner) to the dyad formed of the two. The limited availability of suitable studies may also reflect the greater difficulty of working with dyads (Kazak, 2001).

There currently exist couple-based interventions for cancer patients and their partners that give limited but helpful benefits (Badr and Krebs, 2013) or have mixed results (Vintilǎ et al., 2019), but analysis of them has shown that they frequently lack any specific theoretical foundation. Since both in the area of psychology and in the broader context of health services and public services it is recognized that efforts to modify behavior work better when interventions have a sound theoretical base (Campbell et al., 2000; Craig et al., 2008), the results of this meta-analysis could be seen as an argument for using the STM approach as the departure point for developing couple-based interventions for those facing cancer. Despite the limitations arising from the small number of studies considered, the results of this meta-analytical synthesis have the advantage of being the product of a process of bringing together the results of a number of individual studies. Give the additional fact that they are in harmony with those obtained in earlier research, they can be used as the basis for advancing some ideas regarding the use of the dyadic approach in psycho-oncological research and practice.

The significant associations found between different forms of dyadic coping and both relationship quality and emotional functioning may be seen as arguments in favor of the development and implementation of dyadic interventions based on the STM for couples facing cancer. The fact that the analyses were carried out separately for different forms of dyadic coping (common, supportive, negative, the communication of stress) makes possible a more precise identification of coping behaviors which could be the subject of interventions, depending on the result of an evaluation of each couple and on the aims in view. In addition, a dyadic coping approach in interventions could be more cost-effective, since the emotional functioning both of patients and of their partners can be addressed. Likewise, these kinds of intervention would have the advantage of using the shared time of both partners, which could be a plus given that partners often plead a lack of available time as a problem.

From the point of view of content, interventions based on the STM could be aimed at psycho-education that could help partners to understand the importance of the communication of stress, of providing support via taking over the duties of the other person, and also the importance of concentrating on finding and implementing solutions together. Interventions could also include the practicing of abilities aimed at increasing the frequency of behaviors associated with positive consequences (supportive dyadic coping, common dyadic coping, the communication of stress) and reducing those associated with negative consequences (negative dyadic coping).

To summarize, the novelty elements highlighted by this paper are the following. This research is the first meta-analysis that studies the relationship between dyadic coping conceptualized according to the STM model and both relationship quality and emotional functioning in couples facing a cancer diagnosis. The obtained results suggest that the relationship previously found in the broader context of different stressors between dyadic coping and relationship satisfaction is maintained in the context of cancer, but these relationships seem to be weaker, which raises the hypothesis of variables that could influence their intensity. Also, it was pointed out that in the case of couples in which there is an oncological diagnosis, there is a significant relationship between common dyadic coping and emotional functioning. All these support the idea that in an oncological context this dyadic process is important both in terms of individual outcomes and in terms of couple outcomes.

## Limitations

One already-mentioned limitation of this research study is the small number of papers included. The small number of studies included in the analysis may be due to several factors. Thus, strict inclusion criteria such as considering only STM-based research contributed to the clear delimitation of the area of interest and the unitary conceptualization of dyadic coping but reduced the number of eligible studies. Also, psycho-oncology has relatively recently turned to the dyadic approach of stress and coping. The small number of studies found can also be explained by the difficulty of recruiting participants when one of the eligibility conditions is for them to form couples. Another limitation has to do with the fact that since the inclusion criteria required studies to have been published in English in peer-reviewed journals, it is possible that relevant dissertations, conference papers, and unpublished studies may have been overlooked.

Most of the studies identified were cross-sectional in type, which highlights the need for more longitudinal studies to help in the understanding of any temporal dynamics of the relationships between dyadic coping and both relationship quality and emotional functioning.

All the studies were carried out in countries with a western type culture, which limits the degree to which the results can be generalized. Analyzing these relationships in other cultural contexts too could help to overcome this limitation.

While the methodologies of the studies included in the analysis were appropriate, in order to improve this aspect future studies should ideally furnish clearer accounts of how they addressed potential sources of bias and explain how they calculated their sample size. In addition, bearing in mind that the rate of refusal to participate in the studies varied substantially and was sometimes high, the methodology of future research studies should ideally focus on ways of overcoming the kind of objections to participation that patients and their partners raise.

## Conclusions

This meta-analysis has shown statistically significant relationships between different forms of dyadic coping and both relationship quality and emotional functioning for both cancer patients and their partners. A knowledge of these relationships may have useful implications for clinical practice; however, given the small number of studies reviewed, the findings should be interpreted with caution. Despite this limitation, the results reported here show that the dyadic approach has a part to play as a research direction in psycho-oncology.

## Data Availability Statement

The original contributions presented in the study are included in the article/supplementary materials, further inquiries can be directed to the corresponding author/s.

## Author Contributions

MV and AŞ contributed to all phases of the article. OT contributed to the design phase of the paper, to the collection and analysis of data. All authors contributed to the article and approved the submitted version.

## Conflict of Interest

The authors declare that the research was conducted in the absence of any commercial or financial relationships that could be construed as a potential conflict of interest.
